# Influence of Y_2_O_3_ Content on Structural, Optical, Spectroscopic, and Laser Properties of Er^3+^, Yb^3+^ Co-Doped Phosphate Glasses

**DOI:** 10.3390/ma14144041

**Published:** 2021-07-20

**Authors:** Karel Veselský, Vilma Lahti, Laeticia Petit, Václav Prajzler, Jan Šulc, Helena Jelínková

**Affiliations:** 1 Faculty of Nuclear Sciences and Physical Engineering, Czech Technical University in Prague, Břehová 7, 115 19 Prague, Czech Republic; jan.sulc@fjfi.cvut.cz (J.Š.); helena.jelinkova@fjfi.cvut.cz (H.J.); 2 Photonics Laboratory, Tampere University, Korkeakoulunkatu 3, 33720 Tampere, Finland; vilma.s.lahti@tuni.fi (V.L.); laeticia.petit@tuni.fi (L.P.); 3 Faculty of Electrical Engineering, Czech Technical University in Prague, Technická 2, 166 27 Prague, Czech Republic; vaclav.prajzler@fel.cvut.cz

**Keywords:** erbium Er^3+^, ytterbium Yb^3+^, yttrium, glass, phosphate, spectroscopy, laser

## Abstract

The influence of the addition of Y_2_O_3_ on the structural, spectroscopic, and laser properties of newly prepared Er, Yb-doped strontium-sodium phosphate glass was investigated. While the addition of Y_2_O_3_ has a small influence on the absorption spectra and fluorescence lifetime, it has a strong impact on the emission cross-section and on OH^−^ content. The glasses were used as the active medium for diode-pumped laser emitting at 1556 nm. The increase in Y_2_O_3_ content leads to a significant 35% increase in laser slope efficiency up to 10.4%, but at the expense of the substantial reduction of the wavelength tunability from 82 to 54 nm.

## 1. Introduction

The trivalent erbium (Er^3+^)-doped solid-state glass lasers and amplifiers, operating on the ^4^I_13/2_ → ^4^I_15/2_ transition, are well-known and reliable sources of “eye-safe” laser radiation around 1550 nm wavelength, which is located within the ultra-low-loss telecommunication window of glass [1,2]. Emission in this wavelength range has a wide range of applications, such as range-finding [3], remote sensing [4], medicine [5,6], optical communications [7,8], and others [9]. However, Er^3+^-doped glasses have a low absorption at ~970 nm. For efficient diode pumping, the Er^3+^ ions are usually codoped with Yb^3+^ ions, which act as sensitizers, greatly enhancing the pumping process. The optimum Er^3+^/Yb^3+^ ratio also helps to reduce Er^3+^ ion cluster formation, known to lead to non-radiative energy transfer and losses [10].

There are many varieties of Er^3+^ glasses, such as phosphate, fluoride, silicate, borate, and tellurite glasses, reported in the literature [11]. The phosphate glass system is considered one of the best active glass systems for the preparation of Yb^3+^ sensitized glass laser at 1.5 µm due to its high stimulated emission cross-section, wide UV-VIS-IR transmittance band, low nonlinear refractive index, small up-conversion losses, and low probability of energy back transfer from Er^3+^ to Yb^3+^ [12,13]. Phosphate glass can be designed to have good chemical durability and high rare-earth ions solubility, which reduces the detrimental clustering of rare-earth ions, although it exhibits rather low thermal conductivity. Thus, lasers from phosphate glass are usually driven at low repetition rates; nevertheless, the CW regime can be achieved [14,15].

It is well known that the structural, spectroscopic, and laser properties of rare-earth ion-doped glasses depend on the glass composition and fabrication process. The surrounding ligand field has a considerable influence on the shape of the spectral bands and on basic parameters such as absorption and emission cross-sections, excited-state lifetime, and thus on the overall quantum efficiency of the laser system. Multi-component phosphate glasses can promote the above-mentioned advantages of phosphate glass according to the desired application [15,16,17]. Thus, the interest in the development of new phosphate glasses can be also evidenced by the large number of studies that have been carried out on different types of Er-Yb-doped phosphate glasses, including sodium-aluminum [18], sodium-boron [19], sodium-titanium-strontium [20], zinc-aluminium [21], tellurofluoro [22], lithium-lanthanum [23,24,25], potassium-barium-aluminum [26,27,28], and aluminum-zinc-lead-alkali [29]. Moreover, the optimum Er^3+^-Yb^3+^ concentration and phosphate content have been studied [10,15,23,30]. Er^3+^-doped glasses in the P_2_O_5_-SrO-Na_2_O system have been intensively investigated [20,31], and we demonstrated that the spectroscopic properties of the glasses can be impacted by adding a small amount of Al_2_O_3,_ TiO_2_ or ZnO in the glass network. Although Singh et al. reported that it is possible to increase the thermal stability of an aluminosilicate glass by replacing Al_2_O_3_ with Y_2_O_3_ [32], we found no studies on understanding the impact of Y_2_O_3_ addition on the spectroscopic and laser properties of Er^3+^,Yb^3+^-doped phosphate glass.

In this paper, the influence of the Y_2_O_3_ addition on the structural, thermal, spectroscopic, and laser properties of new sodium-strontium-phosphate glass is presented. The influence of Y_2_O_3_ content on the absorption and emission spectra as well as on the fluorescence decay time is discussed. Under diode pumping, the lasing action was achieved, and the performance of all samples was compared.

## 2. Materials and Methods

**Figure 1:**
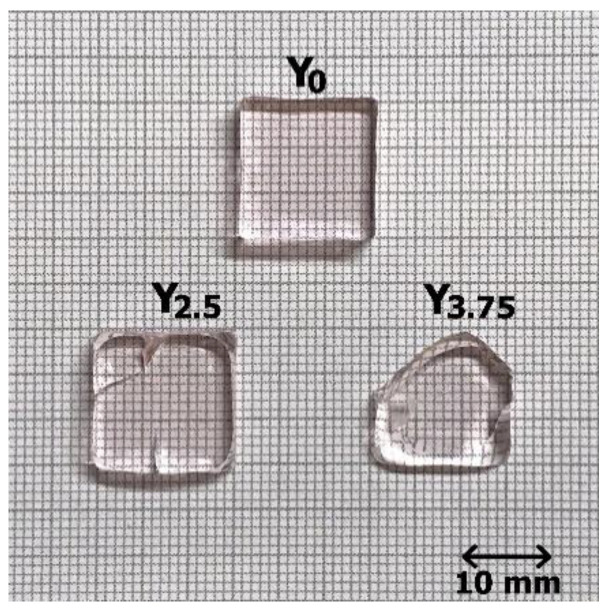
The photo of the investigated Er, Yb:phosphate glass samples. The visible cracks occurred during the polishing of the glasses.

The composition of prepared samples was (98 − x)(0.50P_2_O_5_ − 0.40SrO − 0.10Na_2_O) − 0.5Er_2_O_3_ − 1.5Yb_2_O_3_ − xY_2_O_3,_ (in mol%), where x = 0, 2.5, 3.75 (glasses are labeled as Y_0_, Y_2.5_, and Y_3.75_). The investigated samples were prepared using a melting process. The used raw materials were NaPO_3_ (Alfa Aesar), SrCO_3_ (Sigma-Aldrich, St. Louis, MO, USA, ≥99.9%), Er_2_O_3_ (MV Laboratories Inc., Frenchtown, NJ, USA, 99.999%), Y_2_O_3_ (Sigma-Aldrich, ≥99.99%). Sr(PO_3_)_2_ was prepared at 850 °C using SrCO_3_ and (NH_4_)_2_HPO_4_. Pt crucible was used to melt the 15 g batches. The melting was from 1050 °C to 1500 °C, depending on the glass composition. After 30 min, the glasses were quenched and annealed at 40 °C below their respective glass transition temperatures (T_g_). After annealing, the glasses were polished in the shape of a block with plane-parallel polished faces with a dimension of 15 mm × 15 mm and a thickness of 4.3 mm. The picture of the samples is shown in Figure 1. The slightly pink coloring is due to Er^3+^ doping. The samples were tested without any face coating.

The density (*ρ*) of the investigated glasses was measured using the Archimedes method. Ethanol was used as the immersion liquid. The accuracy of measurement was ±0.02 g/cm^3^_._

The thermal characteristics were determined by the SDT Q600 thermal analyzer (TA instruments) using differential thermal analysis (DTA). The heating rate was 10 °C/min. The glass transition temperature (*T_g_*) was taken at the inflection point of the endotherm, the crystallization temperature (*T_p_*) at the maximum of the exothermic peak and *T_x_* at the onset of the crystallization peak. The accuracy of the measurement was ±3 °C.

The IR absorption spectra were measured using the Perkin Elmer Spectrum FTIR2000 spectrometer. The transmission spectra were measured from bulk glasses in the 2500–4000 cm^−1^ range and with a spectral resolution of 4 cm^−1^, while the absorption spectra in the 650–1500 cm^−1^ region were collected using the Attenuated Total Reflection (ATR) mode from glasses crushed into powder. The spectra were recorded with a resolution of 2 cm^−1^ and 8 scan accumulation.

The absorption spectra in 180–3000 nm were measured by the SHIMADZU UV-3600 spectrophotometer with a spectral resolution of 1 nm. The absorption coefficient *α* was calculated from transmission spectra corrected for Fresnel losses and sample length. The absorption coefficient (*α*) was used to estimate the absorption cross-section (*σ_abs_* (*λ*)) using the following equation [1]:

(1) $$\sigma_{a}\left(\lambda \right)=\frac{\ln\left(\frac{I_{0}}{I}\right)}{N\cdot L}=\frac{\alpha }{N}$$

where ln(*I*_0_/*I*) is the absorbance, *N* is the rare earth ion concentration (ions/cm^3^), and *L* is the sample thickness (cm).

The index of refraction was measured by the Metricon Model 2010/M Prism Coupler refractometer at six different wavelengths with ±0.0005 precision. A detailed description of the measuring method can be found elsewhere [33].

The emission spectrum and lifetime were measured simultaneously at room temperature. The samples were excited by a laser diode at 976.5 nm. The fluorescence radiation was collected from the sample by a parabolic gold mirror Thorlabs MPD229-M01 (reflective focal length of 50.8 mm) and focused into the optical fiber, which was connected to the spectrometer Ocean Optics NIR-512 (spectral resolution of 3.5 nm). The fluorescence decay time was measured by a confocal method using a pair of achromatic doublet lenses (AC508-075-B and AC508-150-B by Thorlabs Inc., Newton, NJ, USA), with a diameter of 50.8 mm and focal length of 75 mm and 150 mm, respectively, and 100 μm pinhole. This setup was used to minimize the influence of reabsorption in the sample. We have used this method successfully for the Yb:YAG and Ho:YAG crystal samples investigation [34,35]. To collect the signal, the InGaAs FGA10 (900–1700 nm) photodiode connected to Tektronix TDS3052B (500 MHz, 5 GS/s) oscilloscope was used. The Si plate was placed before the pinhole to suppress lower wavelength radiation, including pumping. The fluorescence lifetime was obtained by fitting the fluorescence decay time with a single exponential function.

The emission cross-sections were calculated from measured fluorescence intensity spectra and fluorescence lifetime using the Füchtbauer–Ladenburg equation [36]:

(2) $$\sigma_{e}\left(\lambda \right)=\frac{\lambda^{5}}{8\pi \tau cn^{2}}\frac{I\left(\lambda \right)}{\int I\left(\lambda \right)\lambda d\lambda }$$

where *λ* is wavelength, *τ* is fluorescence lifetime, *c* is speed of light in vacuum, and *I(λ)* is fluorescence intensity.

**Figure 2:**
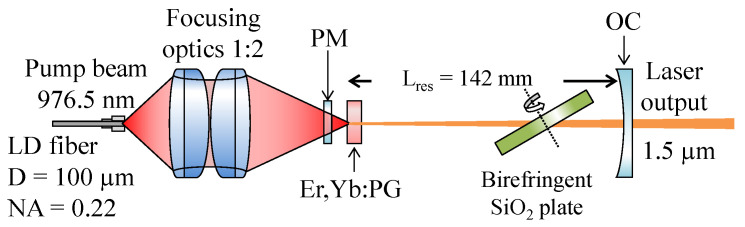
The experimental arrangement of a tunable laser based on the investigated glasses.

The lasing performance was measured using the experimental setup shown in Figure 2. The samples were mounted in a copper holder cooled with tap water (~13 °C). The LIMO35-f100-DL976-EX1202 laser diode (fiber core diameter of 100 μm, NA = 0.22) was used for pumping at 976.5 nm. The AC508-075-B and AC508-150-B lenses forming 1:2 imaging were used to focus the pump radiation into the sample. To prevent damage to the samples, the pumping diode was operated in the pulse regime (f = 10 Hz, pulse duration Δt = 2 ms) with a low duty cycle of 2%. A 142 mm long semi-hemispherical laser resonator was used. The resonator consisted of flat pump mirror PM (HR @ 1.55 µm and HT @ 0.976 µm) and curved output coupler OC (reflectivity R = 98% @ 1.5 µm, r = −150 mm). The pulse duration was measured using the TDS3052B oscilloscope. The absorbed pumped power and laser output power characteristics were measured using a laser power meter Thorlabs S405C. The absorption of pump power was measured for the laser threshold incident power on glass under nonlasing conditions and was used for the calculation of absorbed pump power in the whole applied range. The output power amplitude was estimated from the mean output power, using the known pulse duration and repetition rate. The laser threshold and slope efficiency were calculated using a linear fit. The wavelength of the output radiation was measured with StellarNet DWARF-Star NIR (spectral resolution of 1.25 nm). The output laser beam transverse profile was measured by the Spiricon PYROCAM IV.

## 3. Results and Discussion

### 3.1. Physical and Thermal Properties of the Glasses

The physical and thermal properties of the investigated glasses are summarized in Table 1. The progressive addition of Y_2_O_3_ into the glass increases its density due to the heavy Y atoms, which partially replace P, Na, and Sr in the glass network. The increase in the Y_2_O_3_ content also increases the characteristic temperatures of the glass. The rise in the glass transition temperature (*T_g_*) might suggest that the introduction of Y_2_O_3_ in the phosphate network increases the strength of the network. Moreover, Table 1 also lists Δ*T*, the temperature difference between the onset of the crystallization temperature (*T_x_*) and *T_g_*, which is an indicator of the glass resistance to crystallization. The increase in Δ*T* with an increase in *x* indicates that the addition of Y_2_O_3_ strengthens the thermal stability of the glass against the crystallization.

### 3.2. Structural Properties of the Glasses

**Figure 3:**
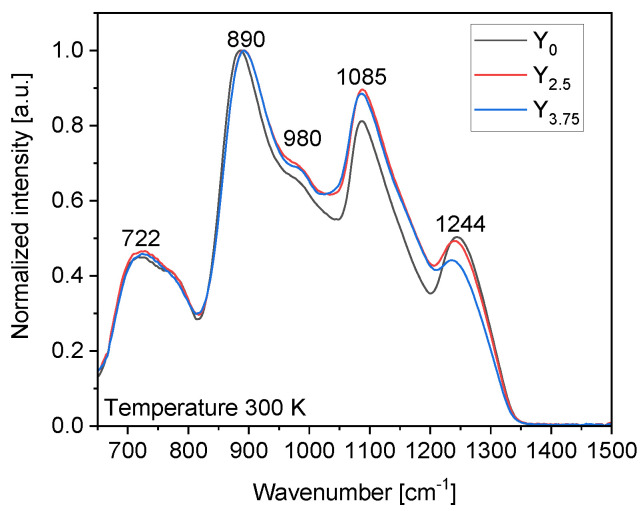
The IR spectrum of the investigated glasses.

The IR spectra of the glasses are shown in Figure 3. They are normalized to the band at 890 cm^−1^. The spectra show bands at ~722, 890, 980, 1085, and 1244 cm^−1^. The IR spectra are similar to those of phosphate glasses. They indicate that the investigated glasses have a metaphosphate structure [20]. The band at ~722 cm^−1^ has been assigned to the symmetric vibrational modes ν_sym_ (P−O−P) of Q^2^ units and the band at 890 cm^−1^ to the asymmetric stretching vibrational modes ν_as_ (P−O−P) in Q^2^ units [37]. The band at 1250 cm^−1^ associated with the shoulder at 1160 cm^−1^ can be related to the asymmetric and symmetric vibrations of PO_2_^−^ in Q^2^ units, respectively [37,38,39], whereas the band at 1085 cm^−1^ with the shoulder at 980 cm^−1^ can be assigned to the asymmetric and symmetric stretching vibrations of Q^1^ units, respectively [38,39].

An increase in the intensity of the band at 1085 cm^−1^ and a decrease in the intensity of the band at 1250 cm^−1^ can be observed with an increase in the Y_2_O_3_ content, indicating that the addition of Y_2_O_3_ to the network leads to a progressive depolymerization of the phosphate network associated with an increase in the Q^1^ units at the expense of Q^2^ units. One can notice that the bands shift towards higher wavenumbers with an increase in the Y_2_O_3_ content, which is a clear sign of changes in the chemical bonds’ strength in the glass network.

### 3.3. Optical Properties of the Glasses

**Figure 4:**
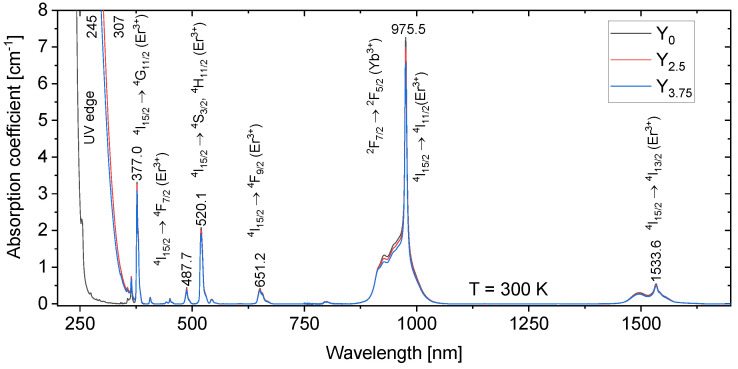
The absorption spectrum of the glasses.

The absorption spectra are shown in Figure 4 and depict the typical absorption bands of Er^3+^ ions. The absorption band at 975 nm corresponds both to the strong 4f−4f transition ^2^F_7/2_ → ^2^F_5/2_ of Yb^3+^ ions and to the relatively weak ^4^I_15/2_ → ^4^I_11/2_ transition of Er^3+^ ions. An increase in the Y_2_O_3_ content leads to a shift of the UV edge from 245 nm to 307 nm, probably due to the depolymerization of the phosphate network.

**Figure 5:**
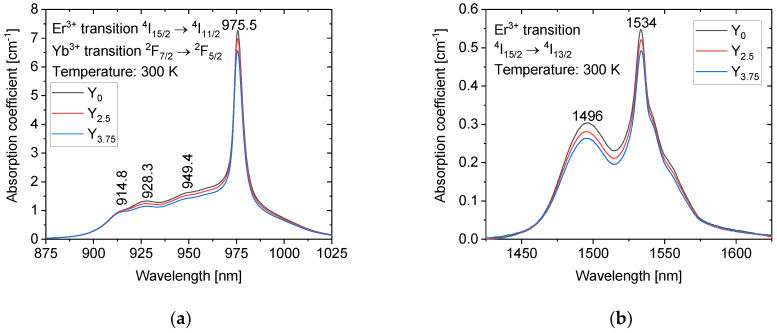
The absorption bands centered at 975 nm (**a**) and at 1534 nm (**b**) of the investigated glasses.

The absorption bands centered at 975 nm and at 1534 nm are shown in Figure 5a,b, respectively. The width of 6.5 nm (FWHM) of the absorption band at 975 nm is independent of the Y_2_O_3_ concentration, and the absorption line is suitable for diode pumping. The maximum value of absorption coefficient at 975 nm is 7.3 cm^−1^ for the glass Y_0_. This absorption band is widened down to 900 nm by several weak absorption lines (914.8, 928.3, and 949.4 nm) with an absorption coefficient of about 1.5 cm^−1^_._ As shown in Figure 5b, the absorption coefficients at 1496 nm and 1534 nm are 0.3 cm^−1^ and 0.55 cm^−1^ for the glass Y_0_, respectively. While the shape of this absorption band remains unchanged, a slight decrease in the absorption coefficient can be seen with an increase in the Y_2_O_3_ content. Based on Figure 5a,b, the sites of the Er^3+^ and Yb^3+^ ions are not expected to be strongly affected by the change in the glass composition. The Er^3+^ and Yb^3+^ ions are considered surrounded mainly by P, Na, and Sr.

The absorption cross-sections at 975 nm and at 1534 nm were calculated using Equation (1) and were found to be (9.33 ± 0.05) × 10^−20^ cm^2^ and (0.70 ± 0.05) × 10^−20^ cm^2^, respectively, for the glass Y_0_, and (8.39 ± 0.05) × 10^−20^ cm^2^ and (0.63 ± 0.05) × 10^−20^, respectively for the glass Y_3.75_. Within 10%, the absorption cross-section remains unchanged as the Y_2_O_3_ content increases, confirming that the sites of the rare-earth ions are not strongly impacted by the changes in the glass composition.

**Figure 6:**
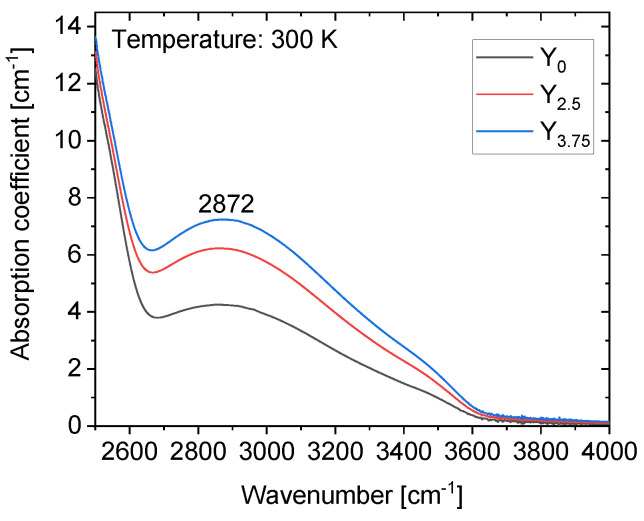
The IR spectrum of the investigated glasses.

The IR absorption spectra of the investigated glasses are shown in Figure 6 and exhibit the typical bands related to the “free” “weakly associated” OH groups at 3500 cm^−1^, the “strongly associated” OH groups at 2800 cm^−1^ and the “very strongly associated” OH group at 2350 cm^−1^ [40]. The absorption coefficient between 2250 and 3750 cm^−1^ increases with an increase in the Y_2_O_3_ content. The increase in the concentration of the OH with the incremental introduction of Y_2_O_3_ to the phosphate glass is suspected to be due to the progressive depolymerization of the phosphate network induced by the addition of Y_2_O_3_. The free OH^−^ content was calculated from the measured absorption coefficient at 2882 cm^−1^ using the following equation [41]:

(3) $$N_{OH}=\frac{N_{A}}{\varepsilon \cdot l}ln\frac{1}{T}$$

where *N_A_* is the Avogadro constant, *l* the glass thickness (cm), *T* the transmittance, *ε* the molar absorptivity of the free OH^−^ groups in the glass, respectively. The molar absorptivity, ε = 49.1 × 10^3^ cm^2^/mol, of silicate glasses [42] was used as no data was found for phosphate glass. The free OH^−^ content increases with the progressive Y_2_O_3_ addition with calculated values of *N_OH_* = 5.2, 7.4, and 8.8 (10^19^ ions/cm^3^) for samples Y_0_, Y_2.5_, and Y_3.75_, respectively. This amount of OH^−^ content is in a similar range that the one reported in phosphate or tellurite glasses [40,43].

The refractive index of the glasses was measured at six different wavelengths: 0.532, 0.6542, 0.8464, 1.3082, 1.5491, and 1.6521 μm with precision ±0.0005 (±0.001 for 1.6521 μm) and fitted with the Sellmeier equation with an infrared correction, which was found to best fit the data in this measurement setup [33]:

(4) $$n^{2}\left(\lambda \right)=A+\frac{B\lambda^{2}}{\lambda^{2}-C}-D\lambda^{2}$$

**Figure 7:**
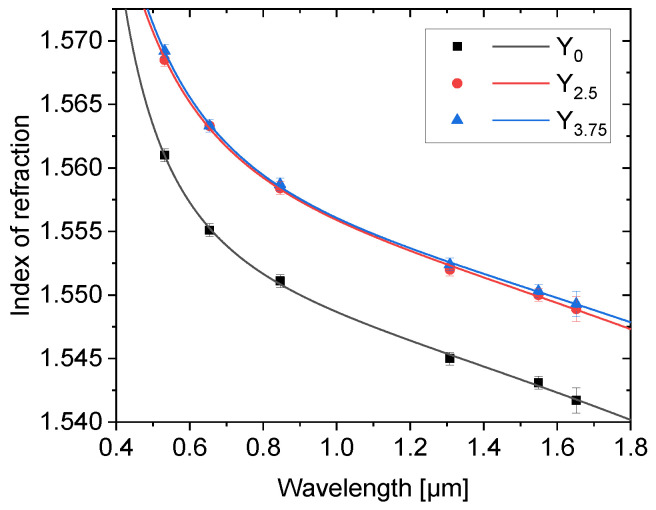
The refractive indices of the investigated glasses fitted with Sellmeier equation with an infrared correction.

The results are shown in Figure 7 and the calculated Sellmeier coefficients are in Table 2.

The refractive indices decreases with increasing wavelength and increases with the addition of Y_2_O_3_ as expected due to the heavy Y compared to the other elements in the glass. The measured values are slightly higher (in the 10^−2^ range) than the average of other Er^3+^:phosphate glasses [10,20,23]. One should notice that the Y_2.5_ and Y_3.75_ glasses exhibit similar refractive indices while having slightly different densities. This can be attributed to the slightly different overall composition of the glass, as explained in [20,44].

### 3.4. Fluorescence Properties of the Glasses

**Figure 8:**
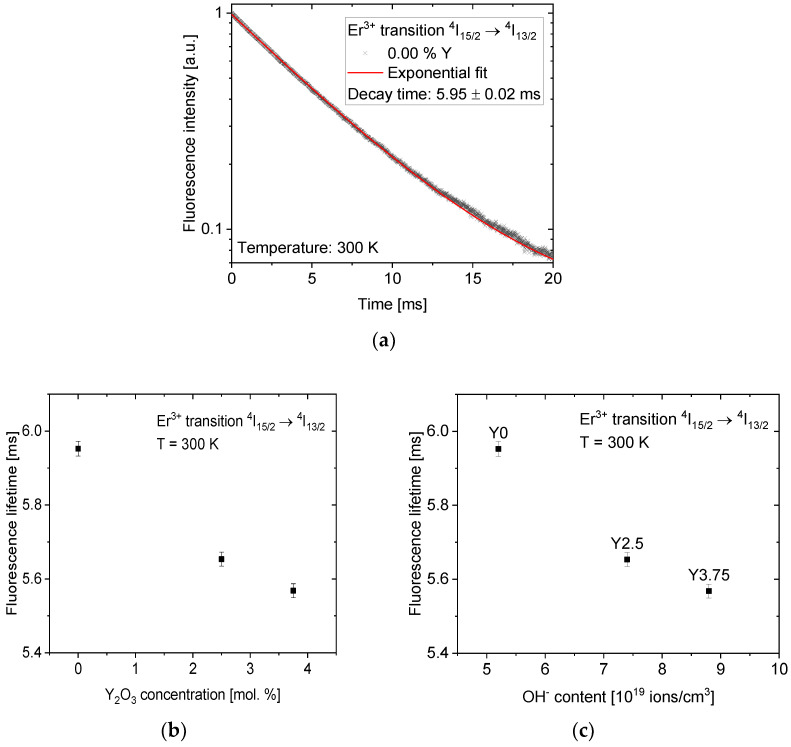
The fluorescence decay curve of the glass Y_0_, taken as an example (**a**). The dependence of fluorescence lifetime of the glasses on Y_2_O_3_ concentration (**b**) and on OH^−^ content (**c**).

The upper-state level ^4^I_13/2_ fluorescence decay curve is shown in Figure 8a, and it exhibits a single exponential behavior for all samples. The fluorescence lifetimes, obtained by fitting the fluorescence decay curve in Figure 8a, are presented in Figure 8b,c.

The fluorescence lifetime value of (5.95 ± 0.02) ms for the glass Y_0_ decreases with the increase in Y_2_O_3_ content down to (5.56 ± 0.02) ms for the glass Y_3.5_. These values are similar to those reported for other phosphate glasses with similar doping concentrations [23,45]. The fluorescence lifetime follows the dependence of free OH^−^ content on Y_2_O_3_ concentration; indeed, the fluorescence lifetime decreases linearly with an increase in the OH^−^ content, as shown in Figure 8c. This relatively weak quenching effect might be explained by the well-known energy transfer from Er^3+^ ions to OH^−^ impurities [45,46,47]. Nonetheless, it is important to mention that the fluorescence lifetime of the investigated glasses is long enough to provide efficient energy storage for Q-switched pulse generation.

**Figure 9:**
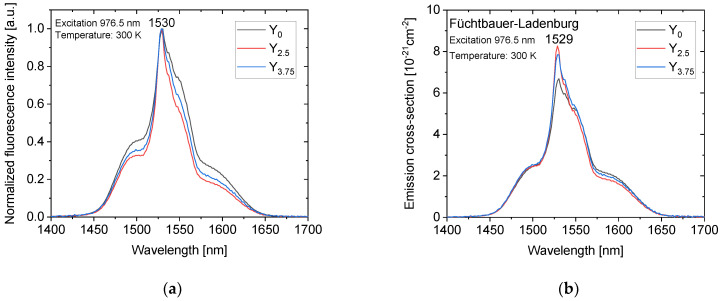
The emission spectra (**a**) and calculated emission cross-section (**b**) of the glasses.

The emission spectra of the investigated glasses corresponding to the laser transition ^4^I_13/2_ → ^4^I_15/2_ are shown in Figure 9a. The fluorescence spectra are smooth and suitable for laser wavelength tuning. One can notice that the addition of Y_2_O_3_ leads to a narrower emission band.

The emission cross-sections were calculated using Equation (2) and are shown in Figure 9b. The emission cross-sections of the newly developed glasses are comparable to those of other Er^3+^-doped phosphate glasses, including commercial ones [13,18,24]. One should notice that both Y-containing glasses exhibit about 20% larger emission cross-section than that of the glass Y_0_. This result follows the dependencies of the refractive index and the fluorescence lifetime and could be a consequence of Y ions causing a partial reduction of Er^3+^ ions clusters and related cooperative upconversion losses due to the depolymerization of the network and thus enhancing the radiative emission [48].

### 3.5. The Laser Performance of the Glasses

**Figure 10:**
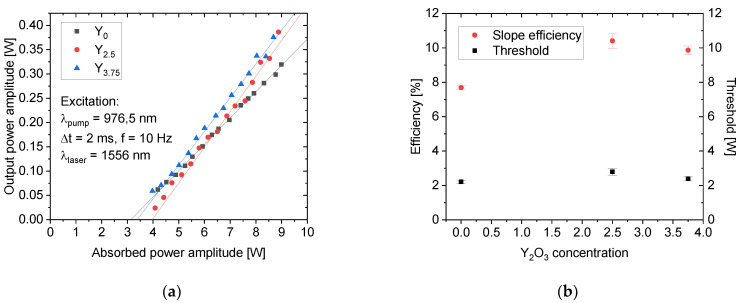
The laser performance of the investigated glasses. The laser output characteristics (**a**) and the laser slope efficiency and threshold in dependence on Y_2_O_3_ concentration (**b**).

The lasing was achieved with all samples with an output wavelength of 1556 nm. To avoid damaging the samples, the pumping diode was operated in the pulse regime resulting in quasi-CW laser output. The measured laser output characteristics with respect to the absorbed pumped power amplitude are shown in Figure 10a.

The slope efficiency of Y_0_ was 7.7%, and it increased with the addition of Y_2_O_3_ (Figure 10b). The best performance was achieved from the glass Y_2.5_ reaching the slope efficiency of 10.4% and the highest output power amplitude of 0.4 W. The laser threshold was 2.2 W for Y_0_ and only slightly increased with Y_2_O_3_ content, revealing the small effect of Y_2_O_3_ concentration on reabsorption and other parasitic processes to laser generation (Figure 10b). Although the overall laser efficiency is about half of what can be achieved with current commercial phosphate glasses [12], the positive influence of Y_2_O_3_ addition can be seen. With further optimization of the fabrication process, the Er^3+^ and Yb^3+^ doping concentration, the length of the active medium, and the addition of antireflection coatings, the increase in the laser performance and efficiency can be expected.

**Figure 11:**
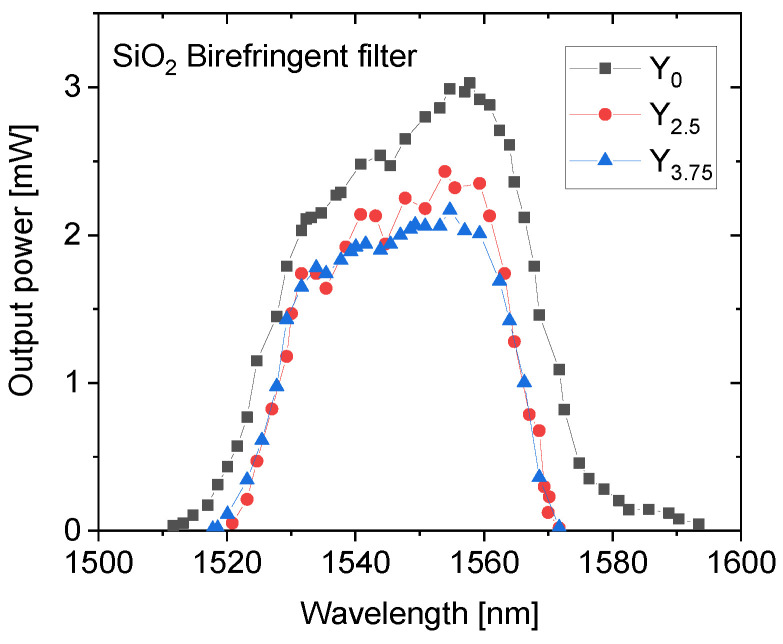
The laser wavelength tuning curves of the glasses.

The tuning of wavelength was achieved with all glasses and the measured tuning curves are shown in Figure 11.

The notches in the tuning curves are due to the absorption lines in the air. The wavelength tuning range of 82 nm (1511–1593 nm) of the Y_0_ sample decreases down to 54 nm (1518–1572 nm) with the addition of Y_2_O_3_. This trend seems to follow the dependence of fluorescence spectra width on Y_2_O_3_ content.

**Figure 12:**
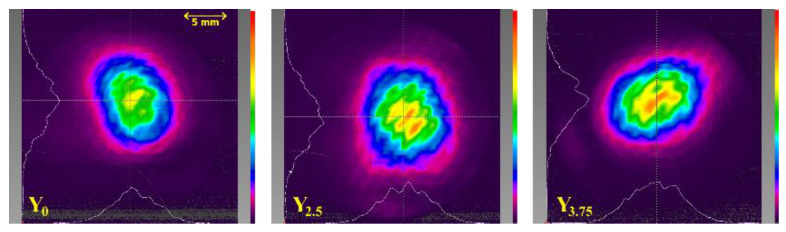
The transverse intensity output beam profiles measured at a distance of 590 mm from OC for maximum pumping.

The captured transverse intensity output beam profiles for maximum pumping are shown in Figure 12. The profiles of all samples are close to Gaussian shape, but they also contain higher-order resonator modes, and the pure basic TEM_00_ mode was not achieved. This could be due to the inhomogeneous structure of the samples and not the optimum overlap of the pump beam with the resonator modes.

## 4. Conclusions

The influence of Y_2_O_3_ content on the structural, physical, thermal, optical, spectroscopic, and laser properties of newly developed multi-component Er^3+^,Yb^3+^ sodium-strontium-phosphate glasses was investigated. Three samples (Y_x_) with molar concentration x = 0, 2.5, and 3.75 of Y_2_O_3_ were studied. The addition of Y_2_O_3_ increases the density and thermal stability against the crystallization of the glass and leads to progressive depolymerization of the phosphate network. It was found that the Y_2_O_3_ content has a minor influence on the absorption spectrum and fluorescence lifetime but a noticeable impact on the emission cross-section and the amount of OH^−^.

The glasses were successfully used as an active medium for the diode-pumped laser with emission of 1556 nm. The increase in the Y_2_O_3_ content leads to a significant enhancement of the laser slope efficiency. Indeed, when adding 2.5 mol% of Y_2_O_3_ in the phosphate glass, the laser slope efficiency can be increased by 35% up to 10.4% with the output power amplitude of 0.4 W but at the expense of the substantial reduction of the wavelength tunability from 82 nm to 54 nm. The positive influence of Y_2_O_3_ addition on laser performance suggests that the desired efficient 1.5 μm diode-pumped laser could be achieved from this new glass system.

## Figures and Tables

**Table 1:** The physical and thermal properties of the glasses. Density ρ, glass transition temperature *T_g_*, onset of the crystallization temperature *T_x_*, crystallization temperature *T_p_*, and supercooled liquid region Δ*T*.

Sample Code	*ρ* ±0.02 (g/cm^3^)	*T_g_* ±3 (°C)	*T_x_* ±3 (°C)	*T_p_* ±3 (°C)	Δ*T* = (*T_x_* − *T_g_*) ±6 (°C)
Y_0_	3.21	456	554	577	98
Y_2.5_	3.26	474	604	629	130
Y_3.75_	3.33	486	626	649	140

**Table 2:** The Sellmeier coefficients (for wavelength in μm) of the investigated samples.

Sellmeier Coefficients	Y_0_	Y_2.5_	Y_3.75_
A	2.239	−136.9821	−42.0888
B	0.157970	139.3971	44.5031
C (µm^2^)	0.059566	0.000096849	0.00031812
D (µm^-2^)	0.0085731	0.0077312	0.0070156

## Data Availability

The data is contained within the article. No dataset presented online.
